# Relationships Between Mental Health, Emotion Regulation, and Meaning in Life of Frontline Nurses During the COVID-19 Outbreak

**DOI:** 10.3389/fpsyt.2022.798406

**Published:** 2022-03-29

**Authors:** Sisi Chen, Wen Zhou, Ting Luo, Lingzhi Huang

**Affiliations:** ^1^Department of Cardiovascular Medicine, The Second Xiangya Hospital of Central South University, Changsha, China; ^2^Clinical Nursing Teaching and Research Section, The Second Xiangya Hospital of Central South University, Changsha, China

**Keywords:** cardiovascular disease nursing, childhood autism Delta virus, nurse, meaning in life, emotional regulation, mental health, Delta virus

## Abstract

**Background:**

The sporadic outbreak of COVID-19 and the constant mutation of the virus have put the public in panic. Frontline nurses' appropriate emotional regulation and mental health are the key to win the victory of fighting against the epidemic. The relationships between these variables directly influence the availability of human resources to combat COVID-19.

**Objective:**

To investigate the relationship between meaning in life, emotional regulation, and mental health of frontline nurses during the Delta virus epidemic.

**Methods:**

A cross-sectional survey was conducted in August 2021 among 105 nurses from the Second Xiangya Hospital, Central South University, Changsha, China, who were deployed at the COVID-19 units in Zhangjiajie People's Hospital. The Chinese Meaning in Life Questionnaire, Emotion Regulation Questionnaire, and Psychological Questionnaire for Emergent Events of Public Health were used to evaluate their meaning in life, emotion regulation, and mental health. Their correlation and the moderating effect of emotion regulation were conducted.

**Results:**

In total, 105 (100%) nurses responded. There were 14 men and 91 women and the mean age was (30.295 ± 4.653) years. The average score of meaning in life and mental health of frontline nurses was 49.971 ± 6.386 and 2.755 ± 2.580, respectively. The meaning in life of frontline nurses was positively correlated with cognitive reappraisal and negatively correlated with expressive suppression and mental health. Mental health was negatively correlated with cognitive reappraisal and positively correlated with expressive suppression. The emotional regulation of frontline nurses has a moderating effect between meaning in life and mental health.

**Conclusion:**

Meaning in life and emotion regulation of frontline nurses were significantly correlated with mental health under the effects of the COVID-19 pandemic. Changing the emotion regulation of frontline nurses, strengthening cognitive reappraisal, and weakening expressive suppression could reduce the predictive effect of meaning in life on mental health.

## Introduction

Since the outbreak of Coronavirus Disease 2019 (COVID-19), it has spread rapidly around the world and has put global public health agencies on high alert (1). The novel corona virus keeps mutating. Delta virus is a variant of SARS-CoV-2, and it has the characteristics of strong transmission ability, short incubation period, high viral load, rapid disease development, and possibly immune escape (2). COVID-19 is not only affecting the mental health of the general population but also that of frontline health care workers. Researches have found a link between mental health of health care workers and outbreaks of COVID-19. These factors include fear of virus exposure and occupational risk, increased working hours and disruption of work-life balance, disruption of family life and support networks, and inability to adapt to a rapidly changing work environment (3, 4).

Nursing is a physically and mentally demanding profession. The COVID-19 pandemic has added additional stress to nurses' daily work and environment, and the COVID-19 pandemic puts forward special requirements on nurses. Nurses need to provide patients with complex nursing service and take measures to prevent the spread of the virus to other patients, their families, and themselves. These unprecedented conditions require nurses to work long hours and to cope with emergency patients and more limited resources, which may lead to burnout (5). Due to the nature of their work, health care workers often work under tremendous pressure. In the context of the epidemic, frontline health workers faced greater pressure and had more negative emotions than before (6, 7). A study of 1,257 health care workers in China (60.8% nurses) found that nurses, women, and frontline health care workers had more significant psychological problems (8). Labrague et al. found that 37.8% of frontline nurses had dysfunctional anxiety (9). A large sample survey of 4,692 frontline nurses found that 42.7% of them had physical symptoms (10), especially headache, sore throat, and lethargy, which were significantly correlated with psychological problems (11). In addition, a cross-sectional study of more than 100 frontline nurses in Wuhan, China, during COVID-19 found that 60 nurses (60%) had poor sleep quality, 46 nurses (46%) had symptomatic depression, and 40 participants (40%) may have anxiety symptoms (12). They had short sleep duration, insomnia symptoms, and significant anxiety and depression (13, 14).

Health care workers faced increased stress, heavy workloads, longer working hours, and insufficient sleep, which were sufficient evidence of burnout (15). A study of burnout among frontline staff in emergency departments at two of Ireland's largest hospitals during COVID-19 found that nearly three quarters (74%) of staff suffered burnout (16). A large study in the United Kingdom found a significant increase in the prevalence of burnout among healthcare personnel in the context of the COVID-19 pandemic, with burnout rates at 79% (17). A similar study conducted among frontline workers in Malaysia also found a 53% increase in burnout among frontline workers (18). However, a study published last year looked at burnout levels among frontline workers in China during the COVID-19 pandemic and unexpectedly found that burnout levels among frontline doctors were significantly lower than they were before and the authors concluded that increased engagement and motivation among frontline workers can reduce burnout (19). Further, multiple losses (such as the restricted business during quarantine; the death of family members and witnessing the death of other individuals; inability to follow death and mourning rituals; and representing the symbolic loss of the individual's way of life, culture, and common social mores) was also one of the main causes of burnout among frontline nurses under the effects of the COVID-19 pandemic. Unexpected death usually makes bereaved individuals have unreal feelings about the loss, probably lasting for a long time, while survivors would strongly need to seek meaning in life (20–23). Studies have confirmed that meaning in life was related to individual's mental health, and the higher the meaning in life, the better the mental health (24).

Meaning in life refers to the individual's perception and experience of the sense of meaning and self-worth of existence (25). In times of crisis, meaning in life has been proved to be a key factor in resilience and coping ability (26). Meaning in life makes individuals feel that life is meaningful and has a clear sense of purpose or mission. Substantial research has indicated that individuals with higher levels of meaning in life are more optimistic and positive (27, 28), have less psychological maladjustment, such as depression and daily troubles (29, 30), and have higher life satisfaction (31). Several studies have also found that individuals with higher levels of meaning in life showed better behavior adjustment (32) and sense of control and were better able to deal with stressful events and negative effects (33, 34). A meta-analysis has also shown that helping adults with severe illness clarify their meaning in life increases their sense of self-efficacy and reduces psychiatric symptoms (35), and individuals exhibit higher levels of resilience and self-efficacy (36). Meaning in life is the primary motivating force for any individual, especially in difficult situations (25). Emotion regulation refers to the way in which individuals re-recognize emotional events or reduce this experience. Individual cognitive evaluation and adjustment play a very important role in alleviating negative emotions (37). So why do individuals with high level of meaning in life have fewer psychological problems, and how do they control and adjust their behavior? However, the major question is whether emotion regulation is linked to these variables, or if there are other variables which play a moderating role in this regard. Therefore, the study hypothesized that meaning in life affects an individual's mental health through emotional regulation.

As the core of the medical rescue team, nursing staff rush to the front to participate in COVID-19 prevention and control work. According to statistics, nursing staff accounted for about 70% of the medical team participating in the anti-epidemic work, and became the main force in the treatment work. Therefore, good physical and mental health of nursing staff is the key to winning the battle against COVID-19. In addition to the ICU and emergency department, most of the nursing staff were transferred from the general ward, and only 13% of the nursing staff had experience of fighting the epidemic.

The understanding of relationships between emotion regulation, meaning in life, and mental health can contribute to preventive interventions for problems related to the mental health of health professionals. This study intends to take emotion regulation as the entry point and take frontline nurses during the Delta virus epidemic period as the research object to explore the effect of emotion regulation on the meaning in life and mental health, so as to provide reference for the psychological intervention of frontline nurses.

## Materials and Methods

### Study Setting and Population

This study was a cross-sectional study. We delivered the online questionnaire created through Google Forms to participants *via* WeChat groups. The first part of the questionnaire introduces the purpose of this study, and participants are required to sign informed consent before participating in the survey. In this study, 105 nurses in a tertiary general hospital in China who were deployed at the COVID-19 units in Zhangjiajie People's Hospital in China from August 9, 2021 to August 30, 2021 were selected by random cluster method. The investigation time was within the second week (7–14 days) after participating in the frontline work.

Inclusion criteria were as follows: (1) Nurses deployed at the COVID-19 units in Zhangjiajie People's Hospital; (2) Support within 7–14 days; (3) Informed consent and voluntary participation in this study. Exclusion criteria were as follows: (1) Logistics staff; (2) Security access personnel.

The sample size was calculated by Gpower 3.1 software, with effect size 0.3, α = 0.05, 1 – β = 0.8, and bilateral probability values. The sample size required for correlation analysis was 82. A total of 105 (100%) valid questionnaires were collected. There were 14 males and 91 females and the mean age was (30.295 ± 4.653) years.

### Tools

#### General Information Form

The main content includes gender, age, marital status, per capita income of the family, professional title and other social demographic information.

#### Chinese Meaning in Life Questionnaire

The meaning in life questionnaire was developed by Steger et al., which was used to evaluate the level of individual's meaning in life (22). This study adopted the Chinese version of the Meaning in Life Questionnaire revised by Sisi Liu et al. C-MLQ has a total of nine items designed in the form of seven-point Likert scale including presence meaning in life and search for meaning in life two dimensions. The presence meaning in life (MLQ-Q) mainly refers to the degree to which an individual feels whether his life is meaningful or not. The search for meaning in life (MLQ-S) refers to an individual's active degree of searching for meaning of life. From “completely inconsistent” to “completely consistent”, the scores were 1–7, respectively. Except item 2, the scores were all positive. Higher scores taken from the scale indicate increasing levels of meaning in life. Cronbach alpha coefficient of the Scale was 0.71. Cronbach alpha coefficient of MLQ-P and MLQ-S were 0.81 and 0.72, respectively (38).

#### Emotion Regulation Questionnaire

The emotion regulation questionnaire was developed by Gross and John (39). ERQ has a total of 10 items designed in the form of seven-point Likert scale including cognitive reappraisal and expressive suppression two dimensions, which was used to evaluate the frequency of emotion regulation strategies. From “strongly disagree” to “strongly agree”, the score is 1–7 points. Cognitive reappraisal mainly refers to reducing emotional reactions by changing the perception of emotional events. Expressive suppression mainly refers to the reduction of subjective emotional experience by inhibiting emotional expression. The Chinese version translated by Li Wang et al. was used in this study. The retest reliability of cognitive reappraisal and expressive suppression were 0.82 and 0.79, and the internal consistency reliability was 0.85 and 0.77, respectively (40).

#### Psychological Questionnaire for Emergent Event of Public Health

The psychological questionnaire for emergent event of public health was developed by Yan Gao et al. ERQ has a total of 25 items in two parts designed in the form of a four-point Likert scale including depression, neurasthenia, fear, obsessive anxiety, hypochondria—five dimensions. From “nothing” to “severe”, the score is 0 to 3. Higher scores taken from the scale indicate deteriorating level of mental health. The score of each dimension is the total score of each item in this dimension divided by the number of items, that is, the average score of this dimension, and the score ranges from 0 to 3 points. The retest correlation coefficient of PQEEPH five dimensions ranged from 0.401 to 0.920, and the retest correlation coefficient of total score was 0.631. The total Cronbach alpha coefficient was 0.692, and the Cronbach alpha coefficients of the five dimensions ranged from 0.755 to 0.804 (41).

### Statistical Analysis

IBM SPSS Statistics version 23.0 was used to analyze data. Spearman correlation and SPSS PROCESS macro program (version 3.2) were applied to correlation analysis and the moderating effect test.

## Results

### Common Method Deviation Analysis

Harman single-factor test was used to test all the scale original item data. The results showed that there were 11 factors with characteristic roots >1, and the variance of the first factor was 29.918%, which was less than the critical standard of 40%, indicating that there was no common method bias in the data.

### Correlation Analysis Between Meaning in Life, Emotion Regulation, and Mental Health of Frontline Nurses

The meaning in life, mental health, and emotion regulation (cognitive reappraisal and expressive suppression) of frontline nurses meet the normal distribution or approximate normal distribution. The average score of frontline nurses was 49.971 ± 6.386 in meaning in life, 29.238 ± 4.318 in Cognitive reappraisal, 13.076 ± 3.909 in expressive suppression, and 2.755 ± 2.580 in mental health. Pearson correlation analysis showed that frontline nurses' meaning in life was significantly positively correlated with cognitive reappraisal and significantly negatively correlated with expressive suppression and mental health, while mental health was significantly negatively correlated with cognitive reappraisal and significantly positively correlated with expressive suppression (see Table 1).

**Table 1:** Correlation analysis between meaning in life, emotion regulation, and mental health of frontline nurses.

	***x***	***S***	**1**	**2**	**3**	**4**
1. Meaning in life	49.971	6.386	1			
2. Mental health	2.755	2.580	−0.544^*^	1		
3. Cognitive reappraisal	29.238	4.318	0.589^*^	−0.469^*^	1	
4. Expressive suppression	13.076	3.909	−0.284^*^	0.522^*^	−0.387^*^	1

* *p < 0.01*.

### Model Test of Moderating Effects

To further verify the relationship between meaning in life, emotional regulation and mental health of frontline nurses, we took gender, age, per capita household income, one child, marital status, and professional title as the control variables, and meaning in life as the independent variable (X). Emotional regulation (cognitive reappraisal and expressive suppression) was used as the moderating variable (W) and mental health as the dependent variable (Y), respectively. Model 1 in SPSS PROCESS macro program was used to analyze the moderating effects. Data analysis was conducted using the percentile Bootstrap method with bias correction. The sample size was 5,000, and 95% confidence interval (CI) was selected. If 95%CI does not contain 0, it indicates statistical significance.

As shown in Table 2, emotional regulation (cognitive reappraisal and expressive suppression) had significant moderating effects on both the path of meaning in life and mental health. 95% CI of interaction items in the path of “meaning in life → cognitive reappraisal → mental health” ranged from 0.009 to 0.033. 95%CI of interaction items in the path of “meaning in life → expressive suppression → mental health” ranged from −0.031 to −0.006; 95%CI did not include 0, and the differences were statistically significant (*p* < 0.01) (see Table 2).

**Table 2:** The moderating effect of emotion regulation in frontline nurses on meaning in life and mental health.

**Dependent variable**	**Independent variables**	**Coeff**	**se**	***t***	***p***	**LICI**	**ULCL**	**Change of *R^**2**^***	***F***	***P***
Mental health	Constant	7.710	1.546	4.987	0.000	4.641	10.779			
	Meaning in life X	−0.083	0.036	−2.332	0.022	−0.153	−0.012			
	Cognitive reappraisal W	−0.176	0.051	−3.466	0.001	−0.277	−0.075			
	X*W	0.021	0.006	3.476	0.001	0.009	0.033	0.051	12.083	0.001
Mental health	Constant	6.770	1.520	4.455	0.000	3.753	9.787			
	Meaning in life X	−0.131	0.029	−4.611	0.000	−0.188	−0.075			
	Expressive suppression W	0.168	0.046	3.635	0.001	0.076	0.260			
	X*W	−0.019	0.006	−2.928	0.004	−0.031	−0.006	0.033	8.571	0.004

**Figure 1:**
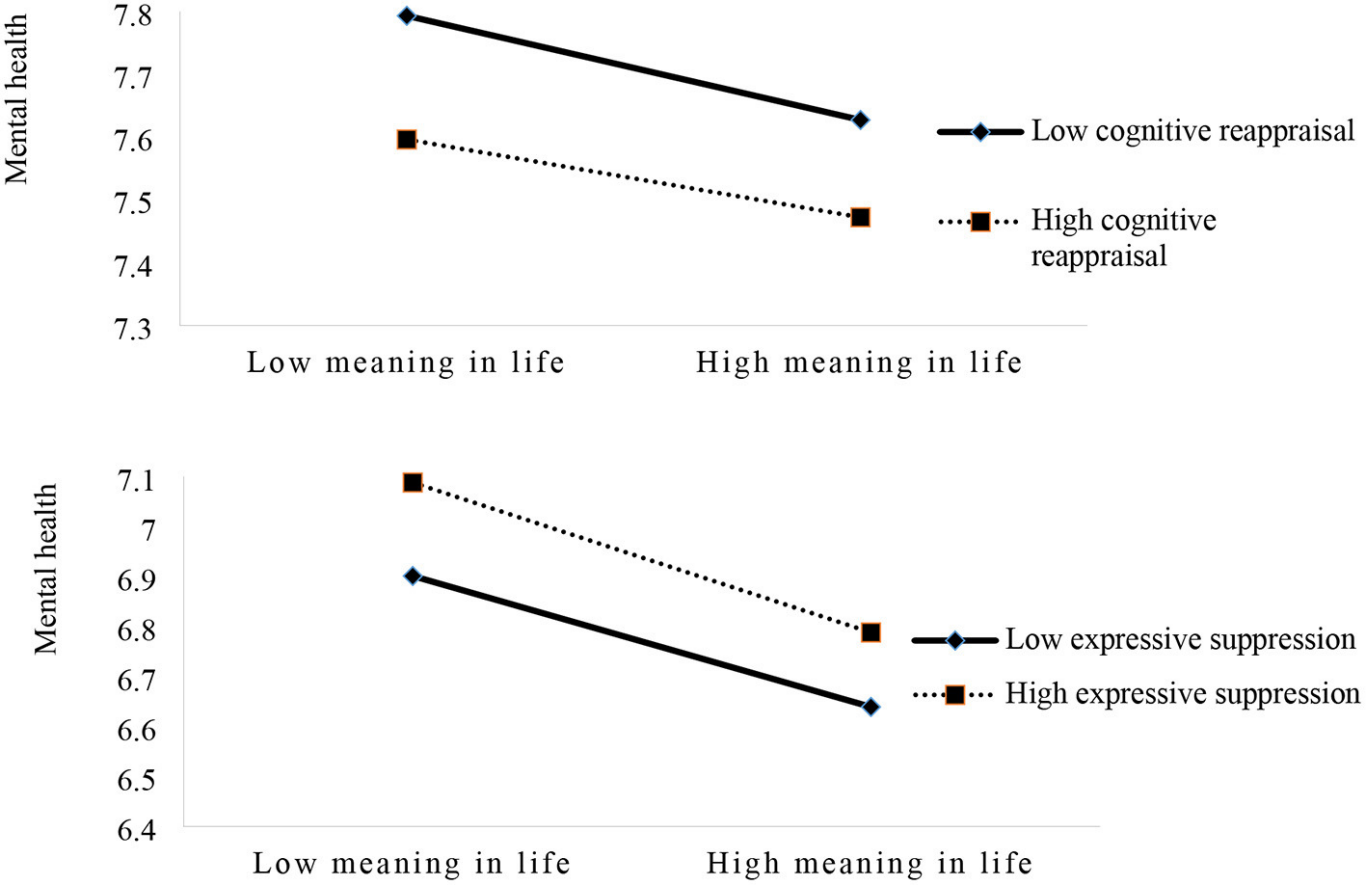
The moderating effect of emotion regulation on meaning in life and mental health.

According to mean ± 1SD (standard deviation), emotion regulation was divided into low emotion regulation group and high emotion regulation group. As shown in Table 3, within a certain range, with the increase of cognitive reappraisal level, the negative predictive effect of meaning in life on mental health gradually weakens. With the increase of the expressive suppression level, the negative predictive effect of meaning in life on mental health gradually increased, indicating that the predictive effect size of low cognitive reappraisal and high expressive suppression was higher (−0.174, −0.203). At different levels of emotion regulation, mental health decreased with the increase of meaning in life (see Table 3; Figure 1).

**Table 3:** The effect size of the moderating effect of emotion regulation style on the different pathways of meaning in life and mental health of frontline nurses.

**Dependent variable**	**Moderating variable W**	**Effect**	**se**	***t***	***p***	**LICI**	**ULCL**
Mental health	W-1SD (Cognitive reappraisal)	−0.174	0.038	−4.535	0.000	−0.251	−0.098
	MEAN (Cognitive reappraisal)	−0.083	0.036	−2.332	0.022	−0.153	−0.012
	W+1SD (Cognitive reappraisal)	0.009	0.049	0.178	0.859	−0.089	0.107
Mental health	W-1SD (Expressive suppression)	−0.059	0.038	−1.550	0.124	−0.135	0.017
	MEAN (Expressive suppression)	−0.131	0.029	−4.611	0.000	−0.188	−0.075
	W+1SD (Expressive suppression)	−0.203	0.037	−5.471	0.000	−0.277	−0.130

## Discussion

The average score of meaning in life and mental health of frontline nurses during the Delta virus epidemic was 49.971 ± 6.386 and 2.755 ± 2.580, indicating that the negative emotions of frontline nurses were not obvious but were more affirmation of self-value and meaning of life, which was consistent with the results of Cai and Gou (42). The results showed that the meaning in life of frontline nurses was significantly positively correlated with cognitive reappraisal and significantly negatively correlated with expressive suppression and mental health. Mental health was significantly negatively correlated with cognitive reappraisal and significantly positively correlated with expressive suppression, which was consistent with the research results of Eastabrook et al. (43) and Yu et al. (44). The results showed that the higher the meaning in life, the better their mental health was if they reduced their emotional response by changing their understanding of emotional events, while the worse their mental health was if they reduced their subjective emotional experience by inhibiting emotional expression. It has been reported that the meaning in life can improve the individual's sense of control over life and provide the individual with a sense of purpose and value (45). People with a high sense of control can better choose effective coping styles and emotional regulation to relieve stress and improve negative emotionality, thus promoting mental health (46). Individuals' emotional regulation affects their mental health by changing the occurrence and process of emotions (47). It suggests that we should pay attention not only to the mental health of frontline nurses but also the adjustment of emotion regulation strategies, improve the meaning in life and cognitive level of frontline nurses, promote the expression of negative emotions, and reduce the occurrence of psychological problems.

The study found that emotional regulation has a moderating effect on the relationship between meaning in life and mental health. In a certain range, with the increase of cognitive reappraisal level, the negative predictive effect of meaning in life on mental health gradually weakened. With the increase of expressive suppression level, the negative predictive effect of meaning in life on mental health gradually increased, and the predictive effect size of low cognitive reappraisal and high expressive suppression was higher. The results showed that the relationship between low cognitive reappraisal and high expressive suppression was stronger than that between high cognitive reappraisal and low expressive suppression and mental health of frontline nurses. Individual mental health and meaning in life are both subjective feelings and experiences (48), while emotion regulation is to re-recognize or reduce such experiences. Studies have confirmed that emotional catharsis is conducive to maintaining a good psychological state, and improving individual cognition can affect individual subjective experience (49). In the process of the impact of environment on people, cognitive assessment and adjustment are always accompanied, and such assessment and adjustment play a very important role in the alleviation of individual negative emotions (8). When the individual's meaning in life is low, the individual lacks the goal and value of life, and the cognitive level is low. The lower the cognitive level is, the more the individual lacks the experience of his own life goals and values and cannot effectively stimulate his own positive psychology, and the more significant the psychological problems are. When the individual carries out cognitive reappraisal of this emotional experience, the psychological problems will be improved accordingly. For individuals seeking meaning in life experience in the process of life, the appropriate containment of their emotional expression can reduce the subjective experience of some bad mood, and mental problems will be reduced accordingly; but when the containment of their events leads to the mood being expressed strongly, emotional experience (positive and negative) is not expressed very well, and mental health will be worse (50, 51). It suggests that we should encourage frontline nurses to express and vent their emotions, improve their cognition of environment and negative events, and improve their emotional experience.

## Summary

In this study, it was concluded that meaning in life and emotional regulation were closely related to the mental health of frontline nurses during the Delta virus epidemic, and emotional regulation plays a moderating role in meaning in life and mental health. This suggests that when we improve the mental health of frontline nurses, we should improve their meaning in life, pay attention to cognitive adjustment and emotional catharsis, and optimize the way of emotional regulation, so as to better improve their mental health level.

## Limitation

Due to the cluster sampling method adopted in this study, the research objects were all nurses in the same hospital, and the sample size was small. There was a large difference in the proportion composition between groups with different demographic variables. Therefore, the extension of the research results should be further discussed by expanding the sample size. This study was a cross-sectional study, and the prediction model of the results may need to be validated by further cohort studies.

## Data Availability Statement

The original contributions presented in the study are included in the article/supplementary material, further inquiries can be directed to the corresponding authors.

## Ethics Statement

All participants fulfilled the informed consent. The study was approved by the Ethics Committee of the Second Xiangya Hospital of Central South University. Ethics Approval No. (2021) and Lunshen No. (Yan 481).

## Author Contributions

TL and LH: conceptualization. SC and LH: data curation. SC: formal analysis and writing—original draft. TL: investigation. WZ and TL: methodology and writing—review and editing. WZ: supervision. SC and TL: software. All authors contributed to the article and approved the submitted version.

## Conflict of Interest

The authors declare that the research was conducted in the absence of any commercial or financial relationships that could be construed as a potential conflict of interest.

## Publisher's Note

All claims expressed in this article are solely those of the authors and do not necessarily represent those of their affiliated organizations, or those of the publisher, the editors and the reviewers. Any product that may be evaluated in this article, or claim that may be made by its manufacturer, is not guaranteed or endorsed by the publisher.
